# Vegetation–environment interactions: plant species distribution and community assembly in mixed coniferous forests of Northwestern Himalayas

**DOI:** 10.1038/s41598-023-42272-1

**Published:** 2023-10-11

**Authors:** Inayat Ur Rahman, Robbie E. Hart, Aftab Afzal, Zafar Iqbal, Rainer W. Bussmann, Farhana Ijaz, Muazzam Ali Khan, Hamid Ali, Siddiq Ur Rahman, Abeer Hashem, Elsayed Fathi Abd-Allah, Ali Sher, Eduardo Soares Calixto

**Affiliations:** 1 Department of Botany, Hazara University, Mansehra, 21300 Khyber Pakhtunkhwa Pakistan; 2 William L. Brown Center, Missouri Botanical Garden, P.O. Box 299, St. Louis, MO 63166-0299 USA; 3 Department of Botany, Khushal Khan Khattak University, Karak, 27200 KP Pakistan; 4 Department of Ethnobotany, Institute of Botany, Ilia State University, 1 Botanical Street, 0105 Tbilisi, Georgia; 5 Department of Botany, State Museum of Natural History, Karlsruhe, Germany; 6 Department of Botany, Bacha Khan University, Charsadda, 24460 KP Pakistan; 7 Department of Biotechnology and Genetic Engineering, Hazara University, Mansehra, 21300 KP Pakistan; 8 Department of Computer Science and Bioinformatics, Khushal Khan Khattak University, Karak, 27200 KP Pakistan; 9 Botany and Microbiology Department, College of Science, King Saud University, P.O. Box. 2460, 11451 Riyadh, Saudi Arabia; 10 Department of Plant Production, College of Food and Agriculture Science, King Saud University, 11451 Riyadh, Saudi Arabia; 11 Department of Agriculture, Bacha Khan University, Charsadda, KP Pakistan; 12 Department of Biology, University of Missouri St. Louis (UMSL), Saint Louis, MO USA; 13 Entomology and Nematology Department, University of Florida, Gainesville, FL USA

**Keywords:** Ecology, Plant sciences, Environmental sciences

## Abstract

One of the main goals of ecological studies is to disentangle the dynamics that underlie the spatiotemporal distribution of biodiversity and further functions of the ecosystem. However, due to many ecological and geopolitical reasons, many remote areas with high plant species diversity have not been assessed using newly based analytical approaches for vegetation characterization. Here, we classified and characterized different vegetation types (i.e., major plant communities) based on indicator species and on the influence of different environmental gradients in the Himalayan mixed coniferous forest, Pakistan. For that, we addressed the following questions: Does the vegetation composition of the Himalayan mixed coniferous forest correlate with climatic, topographic, geographic, and edaphic variables? Is it possible to identify plant communities through indicator species in relation to environmental gradients using multivariate approaches? Can this multivariate be helpful for conservation planning? During four consecutive years we assessed the vegetation composition and environmental variables (21 variables divided in geographic, climatic, topographic, and edaphic groups) of 156 50 m-trasects between an elevation of 2000–4000 m. Using newly based analytical approaches for community characterization, we found a total of 218 plant species clustered into four plant communities with the influence of environmental gradients. The highest index of similarity was recorded between *Pinus-Cedrus-Viburnum* (PCV) and *Viburnum-Pinus-Abies* (VPA) communities, and the highest index of dissimilarity was recorded between PCV and *Abies-Juniperus-Picea* (AJP) communities. Among these four communities, highest number of plant species (156 species) was recorded in PCV, maximum alpha diversity (H’ = 3.68) was reported in VPA, highest Simpson index (0.961) and Pielou’s evenness (0.862) were reported in VPA and AJP. The edaphic gradients (i.e., organic matter, phosphorous, pH and soil texture) and climatic factors (temperature, humidity) were the strongest environmental gradients that were responsible for structuring and hosting the diverse plant communities in mixed coniferous forest. Finally, the Himalayan mixed coniferous structure is more influenced by the spatial turnover beta-diversity process (βsim) than by the species loss (nestedness-resultant, βsne). Our analysis of the vegetation structure along the environmental gradient in the Himalayan mixed coniferous forest supported by sophisticated analytical approaches reveled indicator species groups, which are associated to specific microclimatic zones (i.e., vegetation communities). Within this focus, we side with the view that these results can support conservation planning and management for similar and different areas providing mitigating and preventive measures to reduce potential negative impacts, such as anthropic and climatic.

## Introduction

Ecological studies not only help us comprehend the interaction between vegetation and the environment^1–3^, but they are also required for monitoring global climate change responses^4–6^. Nonetheless, such research identifies vegetation alteration pathways, causes, and processes^7^. Species diversity variation along environmental gradients is a core concern of ecological research^8^, and it has been explained in terms of climate, productivity, biotic interaction, habitat heterogeneity, and history^9,10^. Species diversity is a measure of resilience in ecosystems since it offers resistance to environmental changes^11,12^. Mountains often have a vast altitudinal range, abrupt climatic changes along the altitudinal gradient even over short distances, and a high level of endemism, making them more relevant for such research^13^. Species diversity is reduced as altitude rises due to temperature changes, precipitation, the length of the growth season, changes in solar radiation intensity, chilly, fast winds at high altitude, and steep slope aspect^14^.

Different factors influence the distribution and composition of plant communities in mountainous forests, such as altitude and closely linked edaphic^15^ and climatic factors^16^, topographic heterogeneity^17^, soil chemistry^18,19^, species competition for nutrients^20^, soil texture^21^ and light availability^22^. The environments for species growth and distribution are determined by a combination of these variables. Among these, elevation played a key role in influencing the diversity, richness, and distribution of species^23^. Through run-off redistribution, altitude also affects the availability of soil nutrients and water resources^24^. The moisture regime of dip and scarp landscapes, as well as concave and convex landscapes, differs often, as does the general flora. Run-off accumulates on a range of scales, from little depressions to enormous wades (run-on). As a result, niches and habitats of all types and sizes emerge, dictating the structure and composition of vegetation^25^.

The vegetation within a forest, on the other hand, is strongly affected by the local microclimate^26–28^. Forests have the highest species richness due to the presence of the herb layer^29,30^. By influencing resource availability and environmental variables important to herb layer plants, tree layer diversity can impact herb layer diversity^29,31^. While there have been reports of relationships between herb and tree layer diversity^32^, most research to date have investigated herb layer diversity between forest types in different regions of the Himalaya^33,34^ with only a few dominant tree species or between different monospecific stands, in particular conifer versus broad-leaved forests^29^.

Regarding the effects of environmental gradients (e.g. mountains) on vegetation structure, sophisticated computer-based analyses, such as multivariate analytical programs are very useful to unravel the vegetation structure and dynamic^35^, identifying potential environmental drivers^36–38^ and indicator species^35,39^. For instance, classification and ordination have been used as a way to summarize the multidimensional field data in a smaller number of dimensions clustering similar habitats and stands which share common species^36^. In addition, using these statistical analyses allow us to identity ecological groups, which can be clustered based on indicator values of different environmental drivers such geographic, climatic, topographic, and edaphic.

In this context, the aim of this study was to classify and characterize different vegetation types (i.e., major plant communities) based on indicator species and on the influence of different environmental gradients in the Himalayan mixed coniferous forest, Pakistan. Due to the remote areas with difficult access, uneven terrain, and adverse geopolitical relationships, most part of Himalayan forests has not been assessed using newly based analytical approaches for vegetation characterization. In other words, this study analyzed the vegetation structure and distribution, identifying potential indicator species groups for different microclimatic conditions along the mountainous gradient. Specifically, this study addressed the following questions: Does the vegetation composition of the Himalayan mixed coniferous forest correlate with climatic, topographic, geographic, and edaphic variables? Is it possible to identify plant communities through indicator species in relation to environmental gradients using multivariate approaches? Can this multivariate be helpful for conservation planning? Overall, this study builds toward a better understanding of the systematic description of the plant community of this mountainous region using phytosociological approaches supported using multivariate analyses, which will form the basis for strategic conservation planning.

## Materials and methods

### Study area

**Figure 1:**
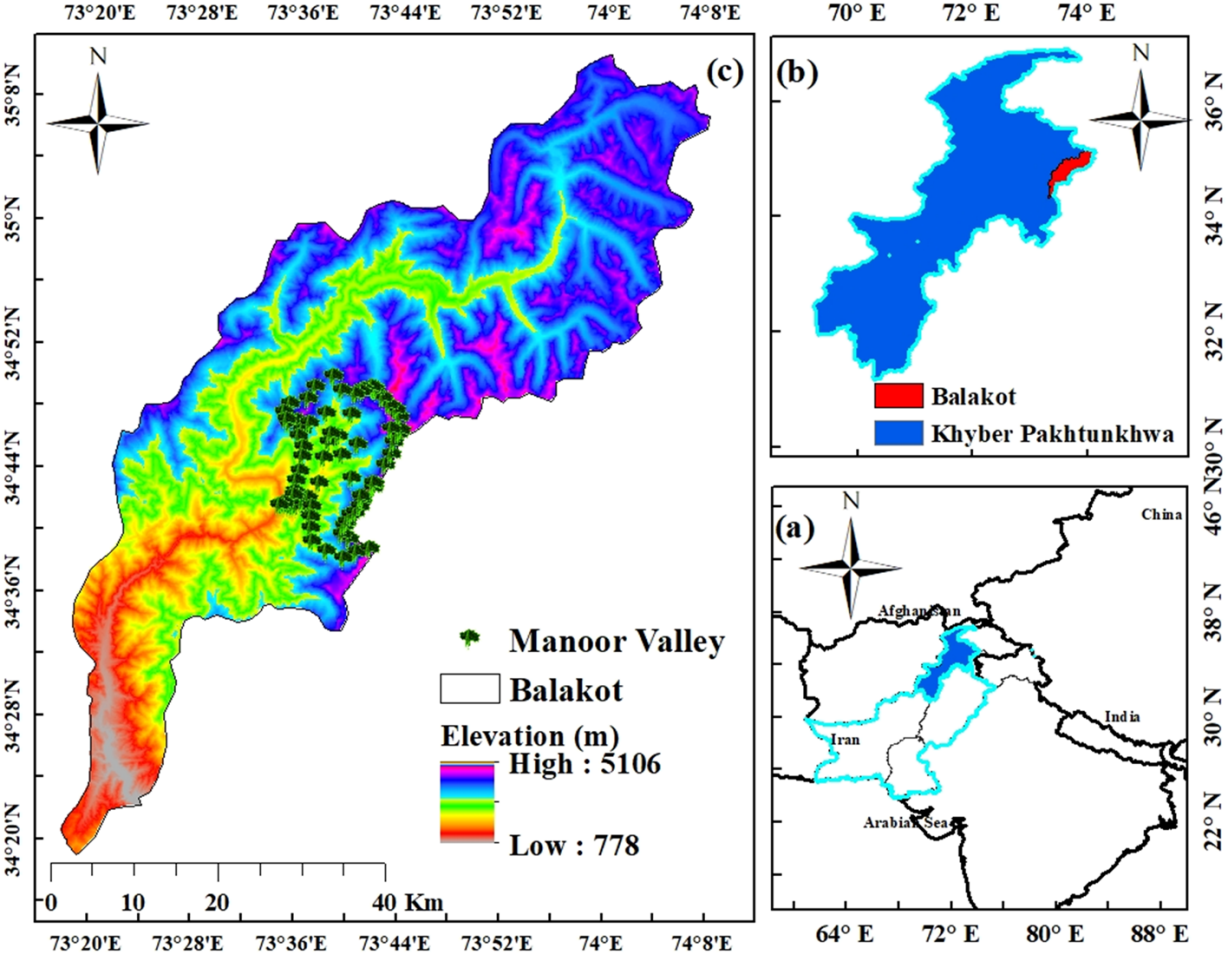
Map of the study area generated through ArcGIS^52^.

The study was carried out in Manoor valley, which is a mountainous valley (34.68165 N to 34.83869 N latitude, and 73.57520 E to 73.73182 E longitude) with 1580 to 4677 m elevation above sea level (Fig. 1) in the Himalayan belt of northwest Pakistan (for more details see Rahman et al.^40–43,46,49,50,122^). The Himalayas are geologically young, having formed when the northward-drifting Deccan Plateau clashed with the Eurasian continent around 50 million years ago, causing geological upheavals that gave rise to the Himalaya, which now extends over 3000 km from Pakistan to Myanmar^44^. Whole area is defined by mountain ranges on both sides of the Manoor river, which runs northeast to southwest along the valley that emerges from Malika Parbat (‘Queen of Mountains,' elevation 5279 m).

The mountains are divided into three ranges, with this ecoregion located in the middle Himalayan range, which reaches a height of roughly 5000 m. Pure fir forest, mixed oak-fir forest, and mixed coniferous forests with fir, blue pine, and spruce are among the forest types found in the ecoregion^44^. The valley has been identified as an important region of the Himalayas with Sino-Japanese vegetation^45^, which is mainly composed of interconnecting mountain ranges that sustain monsoon-driven flora in a drier, colder climate^42,46^. This Himalayan part of Pakistan possesses a rich flora due to the fact that this part of country remained undisturbed by man for a long period; this enabled many species to survive and to evolve^47^. Even across short distances, the flora varies greatly^48^, with a high degree of resource seasonality^49,50,122^ and diversity in both species^42,48^ and communities^51^.

### Vegetation sampling and plant identification

During four consecutive years (2015–2018), 13 sampling sites ranging from 2000 to 4000 m above sea level were selected in the mixed coniferous forest of Manoor valley, Himalaya, Pakistan. Line transect ecological technique was used for vegetation sampling^36^. In each sampling site, three transects of 50 m and 100 m apart each other were determined. In each year of evaluation, three different transects were designed to cover the largest vegetative area of the sampling site, totaling 12 transects per sampling site and 156 transects over the four years of analyses. For each sampling site, relative values of density, frequency, cover and Importance Value Index (IVI) were calculated following the methodology of Curtis and McIntosh^53^ and Buckland et al.^54^. Each sampling site's GPS coordinates were recorded. The clinometer was used to identify the mountain's aspect, which included east (E), west (W), south (S), and north (N), as well as latitude, longitude, and height (GPS). Plant specimens were collected, tagged, placed between newspapers, pressed using a plant presser, poisoned with Mercuric Chloride and Ethyl Alcohol solution, and mounted on herbarium sheets^55,56^. Plant specimens were identified by the expert plant Taxonomists (Dr. Abdul Majid, Prof. Dr. Ghulam Mujtaba Shah and Dr. Jan Alam) of Hazara University, Mansehra, Pakistan. The Flora of Pakistan and other accessible sources were used to identify all of the specimens^57–59^. Scientific name of plant species was cross-checked and updated with an online website (www.theplantlist.org) of the Royal Botanic Gardens, Kew (The Plant List, 2013), accessed on 13 November 2018. Voucher numbers were given to the plant species (Supplementary data Table S1), which were then deposited in the Hazara University Herbarium in Mansehra, Pakistan.

#### Ethical approval

This study was permitted and approved by the “Ethical committees of the Department of Botany” as well as “Advanced Studies and Research Board, Hazara University Mansehra, Pakistan”. Experimental research and field studies on plants (either cultivated or wild), including the collection of plant material, comply with relevant institutional, national, and international guidelines and legislation.

### Environmental gradients

In terms of edaphology, 200–400 g soil samples were taken from three random sampling site in each transect from a depth of 0–30 cm and properly mixed to produce a composite sample^60^. The samples were packed in polythene bags and labelled with a permanent marker. Furthermore, stones and other raw materials were sieved out before the remaining samples were dried in the shade. For each soil sample, physicochemical analyses were performed, including soil texture (clay, sand, silt, loam), pH^61^, electrical conductivity (EC)^62^, organic matters (OM)^63^, nitrogen (N), phosphorus (P), potassium (K), and calcium carbonate (CaCO3) concentrations^64–66^. With the use of a portable weather station (Kestrel weather tracker 4000), several environmental gradients (such as barometric pressure, dew point, humidity, heat index, temperature, wet bulb, and wind speed) were also determined. Here altitude is used as proxy for all the said environmental variables^67,68^.

### Statistical analyses

All the gathered data of species, topographic as well as other environmental variables data were organized to determine the association between them^69,70^. The analyses were conducted using matrices of IV data from all studied sites.

#### Indicator species analysis

TWINSPAN (two-way indicator species analysis) was used to identify plant communities and their primary indicator species using PC-ORD version 5.0.^36^. This analysis applied Sorenson Distance Measurements using Wards Linkage Method^71^ and IVI to identify trends of similarity^72^.

#### Diversity patterns

For each stand, the following diversity patterns were calculated: species richness, Pielou's evenness, Shannon (H'), and Simpson diversity indices. The H' value reveals not only how many species there are, but also how their abundance is distributed throughout all the species in the community. Pielou’s evenness reveals how plant species are evenly distributed within a recognized community. Higher H’ values represent maximum diversity. The relative abundances of the most significant species are particularly sensitive to changes in Simpson's index. Closer to 1 indicates clustering of individuals in a few species, whereas a small number (near to 0) indicates a more equal distribution of individuals across species.

To evaluate which is the beta-diversity component that most influence the vegetation distribution and structure in the area, we used the (1) spatial turnover (Simpson pairwise dissimilarity) and (2) nestedness-resultant components (nestedness-fraction of Sorensen pairwise dissimilarity) of β-diversity applying “Sorensen” as family of dissimilarity index^73–75^. Dissimilarity analysis and dendrograms were conducted in the package “betapart”^76^ and “dendextend”^77^ respectively.

#### Regression models

To compare the parameters (21 parameters distributed into the four different groups: geographic, edaphic, climatic, and slope) evaluated among the communities produced by our previously analyses, we conducted a Generalized Linear Model (GLM) with Gaussian error distribution followed by Likelihood ratio test using the packages “stats” and “car”^78^, respectively. In addition, after calculating species richness, Pielou’s evenness, and Shannon and Simpson diversity for each stand of each plant community, we ran a GLM followed by Likelihood ratio test. In the case of species richness, we used a Poisson error distribution, since we have a count response variable; for others we used Gaussian error.

#### Ordinations

To depict the floristic relationships among the key syntaxonomic units (communities), non-multidimensional scaling ordination (NMDS) and Principal Component Analysis (PCA) were used^79^ using the package “vegan”^80^ in the software R 4.0.0^81^.

To check how explanatory factors (geographic, climatic, edaphic, and topographic) affect plant species distribution, we used canonical correspondence analysis (CCA) and variation partitioning tests (partial CCA)^82^. First, we used the step function with permutation in the "stats" package to create the optimal model with the fewest variables, those that best explain variance^81^. Next, we used the Variance Inflation Factor (VIF) to assess multicollinearity across variables in the final model, and we excluded any variables with VIF > 10 one by one. Finally, we used CCA and partial CCA on the final model to see how much each set of variables explained in our model.

## Results

### Characterization and classification of vegetation

**Figure 2:**
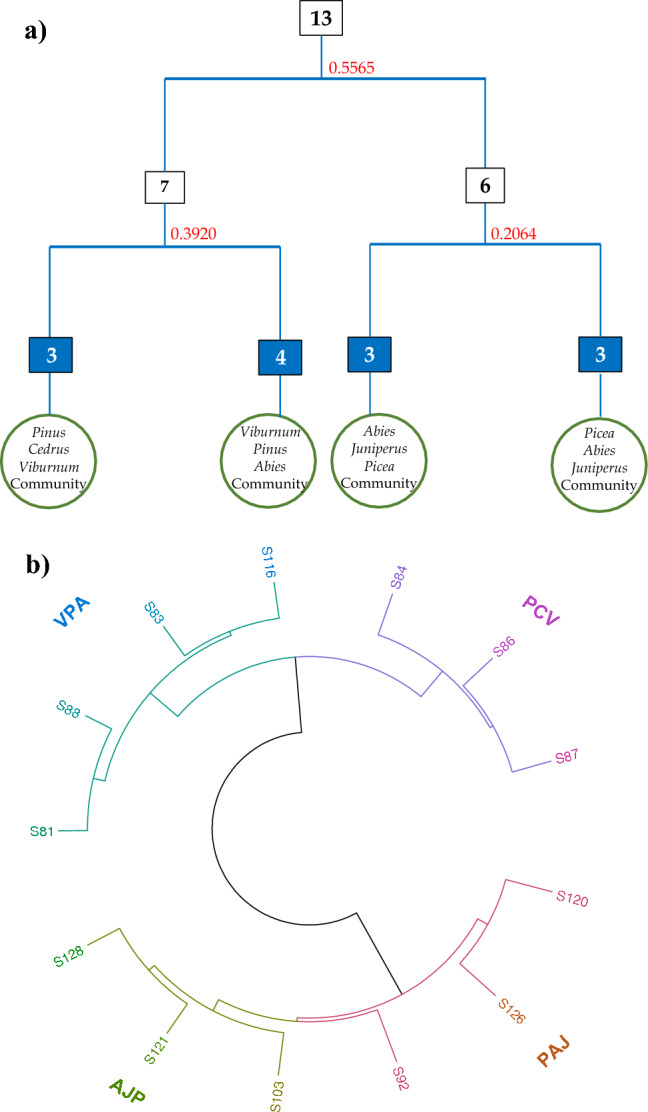
(**a**) Two Way Indicator Species Analysis recognized four major plant communities in mixed coniferous forests of Himalayas. (**b**) A chord diagram of 13 sampling sites with 218 plant species based on Sorenson Distance Measurements presenting 4 major plant communities structured in mixed coniferous forest. PCV, *Pinus-Cedrus-Viburnum*; VPA, *Viburnum-Pinus-Abies*; AJP, *Abies-Juniperus-Picea*; PAJ, *Picea-Abies-Juniperus.*

In the mixed coniferous forest of Manoor valley, Himalaya, Pakistan, 218 plant species were documented from 13 study sites (Supplementary data Table S1). Under the effect of several observed environmental factors, TWINSPAN (Twoway Indicator Species Analysis) categorization identifies four plant communities (Fig. 2). TWINSPAN and indicator species analyses discovered the following communities, which are discussed below.

#### *Pinus-Cedrus-Viburnum* (PCV) community

**Figure 3:**
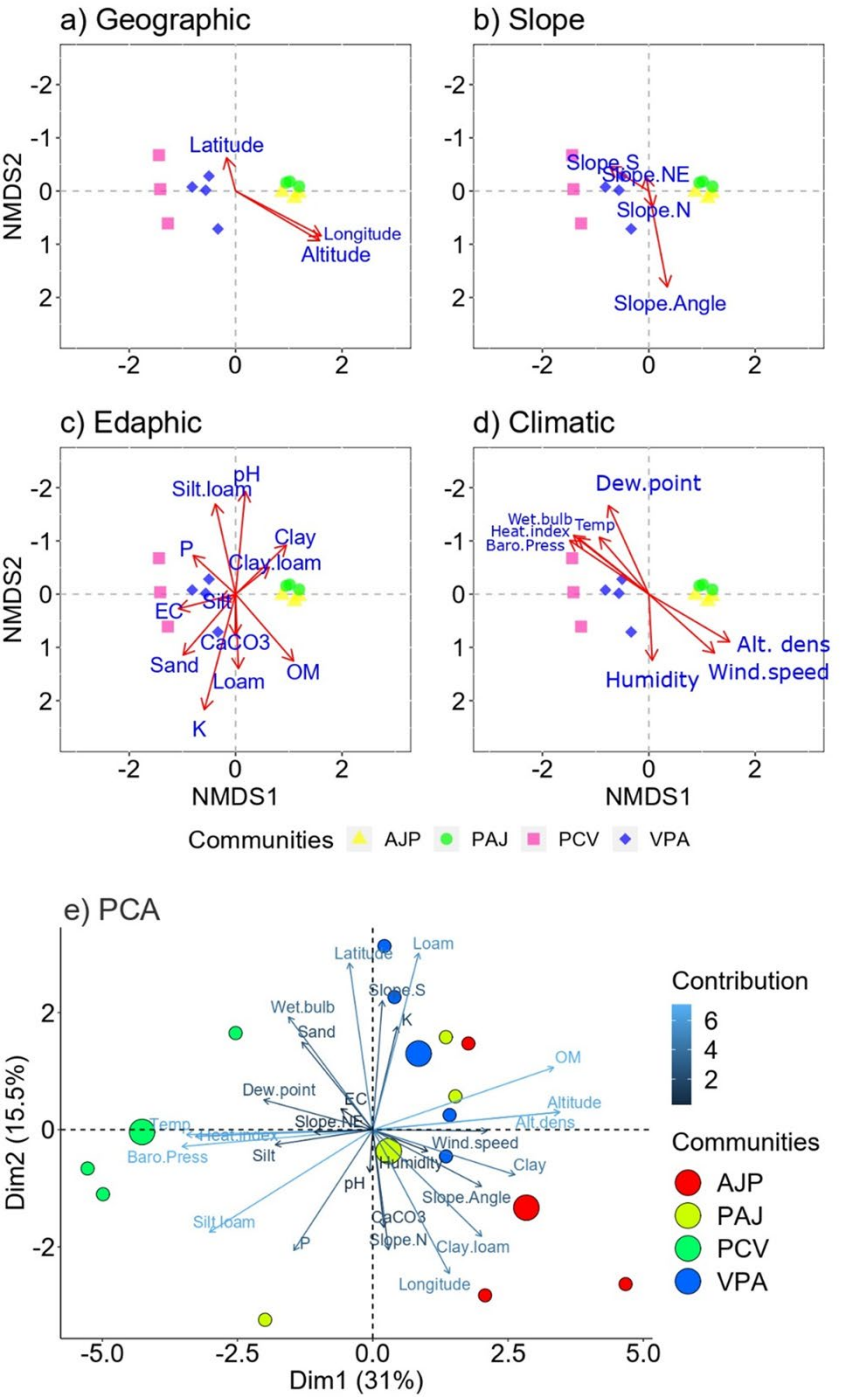
Non-Multidimensional Scaling (NMDS) among plant communities and environmental gradients: (**a**) geographic, (**b**) slope, (**c**) edaphic and (**d**) climatic. (**e**) The association between several measurable environmental factors and the communities depicted in coloured circles using Principle Component Analysis (PCA). Based on a 95% confidence level, each circle with a distinctive colour represents a distinct community. PCV, *Pinus-Cedrus-Viburnum*; VPA, *Viburnum-Pinus-Abies*; AJP, *Abies-Juniperus-Picea*; PAJ, *Picea-Abies-Juniperus*. The length of the arrows indicates the effect and strength of each environmental gradient, while the direction of the arrows indicates the correlation of each environmental gradient. Positive correlation was found for gradients on the same axis, whereas negative correlation was found for gradients on opposing axes. Big circle in (**e**) demonstrates the centroid of each plant community.

This plant community was recognized in three sampling sites located at an elevation range of 2568–2737 m (Fig. 3a), latitude (34.76556 to 34.77056 N) and longitude (73.63194 to 73.64222 E) from northern-east aspect with 37.67º slope exposure (Fig. 3b; Supplementary data Table S2). PCV community was accompanied by 156 plant species. The leading indicators of this major group were *Pinus wallichiana, Cedrus deodara* and *Viburnum grandiflorum* having IVI of 21.28, 17.98 and 16.87 respectively (Supplementary data Table S1). Other associated co-dominant species of the understory community in herbaceous layer were *Arisaema jacquemontii, Cynodon dactylon, Potentilla anserina, Fragaria nubicola, Ranunculus laetus, Urochloa panicoides* and *Hypericum perforatum*. The shrubby layer was dominated by *Rosa brunonii, Jasminium humile* and *Rosa webbiana*. Thus, this community is the representative of mixed coniferous forest thus, recorded in the sloppy surface of the mountains. Edaphic gradients had significant role in structuring this community, i.e., acidic pH (5.33) and low organic matter (0.58–0.92%), higher phosphorous (12.23 mgkg^−1^), moderate potassium (210.82 mgkg^−1^), calcium carbonate (5.49 mgkg^−1^) and electric conductivity (1.87 dsm^−1^) (Supplementary data Table S2). Soil texture of the hosting the community was silty loam with maximum concentration of sand (41.57%) and silt (36.30%), minimum clay (22.13%) in the soil (Fig. 3c). Moreover, climatic gradients had also positive influence in recognition and shaping of this community (Fig. 3d), i.e., higher temperature (19.03 °C), humidity (51.70%), dew point (16.54) and wet bulb (18.87), heat index (21.17), barometric pressure (749.57), and low wind speed (1.67 m sec^−1^).

#### *Viburnum-Pinus-Abies* (VPA) community

A total of four sampling sites hosted VPA plant association along with 128 plant species at an elevational range of 2952–3191 m (Supplementary data Table S2). This community was recorded on North to Southern aspect (Fig. 3b) having the latitude (34.74472 to 34.78111 N) and longitude (73.62083 to 73.65278 E) (Fig. 3a) with 49.50° slope angle. The topmost indicator species of this association were *Viburnum grandiflorum* (16.10 IVI)*, Pinus wallichiana* (15.51 IVI) and *Abies pindrow* (14.85 IVI). Moreover, other most frequent associated herbs of this community were *Arisaema jacquemontii, Bergenia stracheyi, Rheum australe, Fragaria nubicola, Poa infirma* and *Caltha palustris* (Supplementary data Table S1). The shrubby layer was dominated by *Juniperus squamata, J. communis, Cotoneaster microphylous* and *Viburnum cotinifolium*. The tree layer was dominated by *Picea smithiana, Quercus incana, Cedrus deodara* and *Acer caesium*. Furthermore*, Indigofera heterantha, Punica granatum, Poa annua, Ranunculus muricatus* and *Pteris vittata* were recorded as less frequent associated species and *Medicago sativa, Rumex nepalensis, Trifolium repens* and *Inula cuspidata* as rare herbs of the understory community. Soil texture played the influential role in hosting this community was loamy clay with maximum concentration of sand (40.55%) and clay (30.95%), minimum silt (28.50%) in the soil. Moreover, other edaphic gradients structuring the VPA community indicators and distribution of their associated plant species were high organic matter (1.74%), electrical conductivity (2.32 dsm^−1^), calcium carbonate (6.70 mgkg^−1^), potassium (219.53 mgkg^−1^) and phosphorous (11.74 mgkg^−1^) concentrations as compared to other communities (Fig. 3c, Supplementary data Table S2). The climatic gradients responsible for the recognition and shaping of this community were high humidity (57.69%), wet bulb (18.16), minimum temperature (15.55 °C), and moderate wind speed (2.38 m sec^−1^) and barometric pressure (714) (Fig. 3d).

#### *Abies-Juniperus-Picea* (AJP) community

*Abies-Juniperus-Picea* community was hosted by three sampling sites located at an elevation range of 2989–3260 m, latitude (34.709444 to 34.78528 N) and longitude (73.65083 to 73.68639 E) (Fig. 3a) from northern aspect with 55.67° slope angle (Fig. 3b; Supplementary data Table S2). This community was accompanied by 58 plant species. The leading indicator species of this association were *Abies pindrow, Juniperus squamata,* and *Picea smithiana* having the IVI of 24.11, 18.94 and 18.94 respectively (Supplementary data Table S1). The topmost co-dominants of AJP community of herbaceous layer were *Bergenia stracheyi, Thymus linearis, Rheum australe, Sibbaldia procumbens, Poa infirma, Bistorta affinis, Primula hazarica* and *Caltha palustris.* Nonetheless, the dominant shrub species were *Juniperus communis, Cotoneaster microphylous* and *Juniperus excelsa,* and tree co-dominants species were *Pinus wallichiana, Quercus incana* and *Acer caesium.* The understory plant community was hosted by clay-loamy soil with higher clay quantity (36.41%), sand (35.41%) and clay (28.18%). Besides this, AJP community was recorded under the impact of higher organic matter (1.67%), calcium carbonate (6.76) and moderate concentration of electric conductivity (1.72 dsm^−1^). Moreover, lowest potassium (207.60 mg/kg) and phosphorous (10.50 mg/kg) concentrations were recorded in the hosting site of this community (Fig. 3c). Nonetheless, the climatic gradients responsible for shaping of this community were minimum temperature (14.10 °C), heat index (15.13), dew point (14.34), wet bulb (15.39) and barometric pressure (709), and maximum wind speed (3.17 m sec^−1^) (Fig. 3d, Supplementary data Table S2).

#### *Picea-Abies-Juniperus* (PAJ) community

A total of three sampling sites resided this community accompanied by 52 plant species on an elevation range of 2874–3240 m (Supplementary data Table S2). This community was recorded on North to Southern aspect having the latitude (34.73111 to 34.79694 N) and longitude (73.64000 to 73.66750 E) (Fig. 3a) with 46.67° slope angle (Fig. 3b). The chief indicators of this plant community were *Picea smithiana, Abies pindrow* and *Juniperus squamata* with the importance values 28.61, 28.24 and 24.60 respectively. Other co-dominants of the community were *Thymus linearis, Bistorta affinis, Caltha palustris, Rheum australe, Poa infirma, Potentilla argentea, Bergenia* stracheyi, *Androsace hazarica,* and *P. anserina*. The topmost frequent shrubby layer was represented by *Juniperus communis, Cotoneaster microphylous* and *Juniperus excelsa* (Supplementary data Table S1). This community was resided by loamy silt soil texture with higher sandy texture concentration of silt (36.28%) and sand (34.84%), minimum clay (28.88%). Further, edaphology of the understory community comprehends lowest electrical conductivity (1.08 dsm^−1^), calcium carbonate (4.97 mgkg^−1^), potassium (203.29 mgkg^−1^) and phosphorous (11.08 mgkg^−1^), weak acidic pH (5.83), moderate OM (1.44%) concentrations as compared to other communities (Fig. 3c, Supplementary data Table S2). Nonetheless, the climatic gradients responsible for the recognition and shaping of this community were high wind speed (2.50 m sec^−1^), minimum temperature (16.83 °C), wet bulb (16.26), dew point (15.22) and moderate humidity (53.19%) (Fig. 3d).

### Diversity patterns

**Figure 4:**
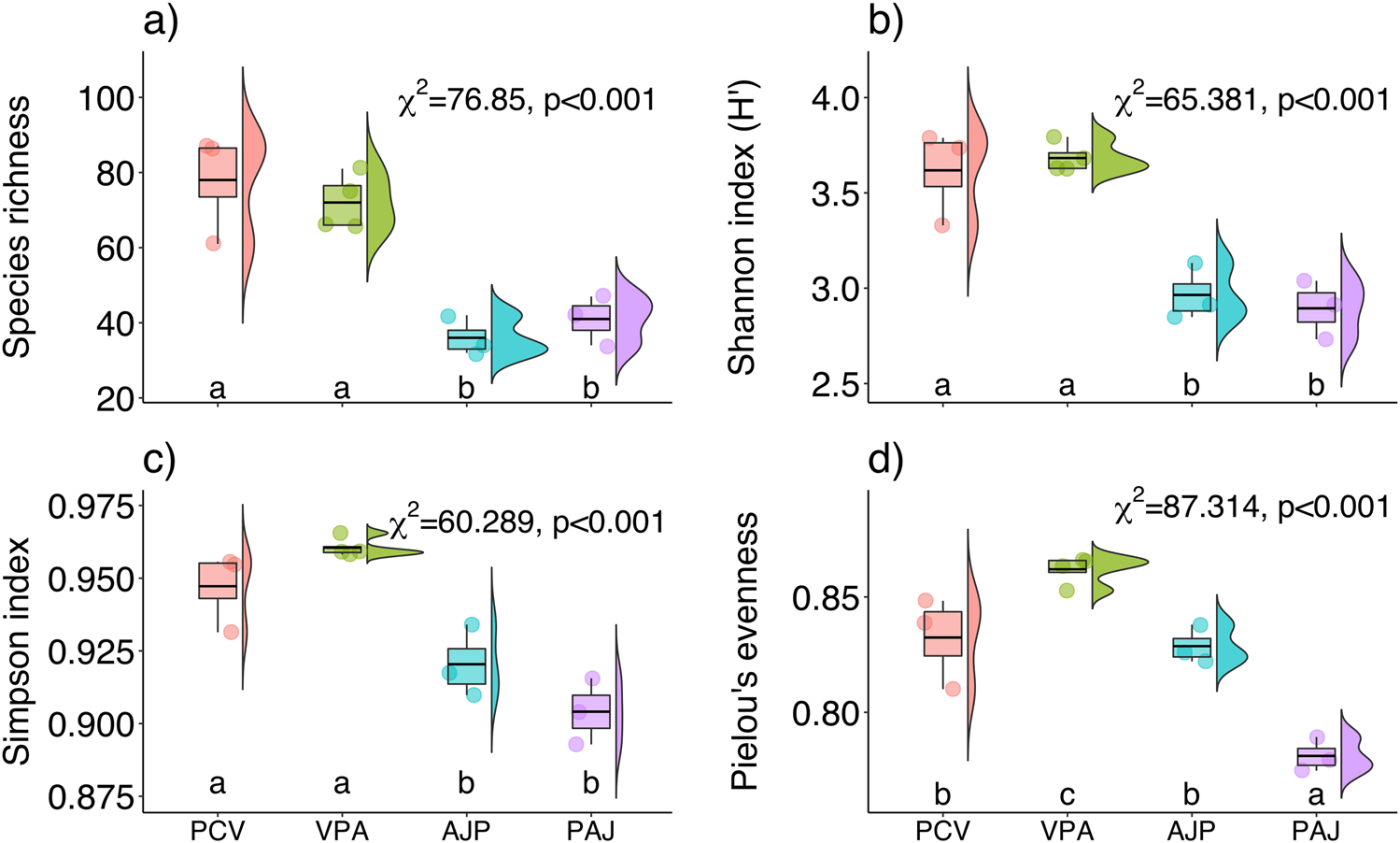
(**a**) Species richness, (**b**) Alpha diversity (Shannon diversity), and (**c**) Simpson's diversity (beta diversity), (**d**) Pielou's evenness in respect to elevational gradient across four plant groups. Plant communities in x-axis were plotted in ascending order according to elevation gradient (low to high elevation). PCV: *Pinus-Cedrus-Viburnum,* VPA: *Viburnum-Pinus-Abies,* PCV: *Abies-Juniperus-Picea,* PAJ:* Picea-Abies-Juniperus.*

Species richness values differed significantly (χ^2^ = 76.85, df = 3, *p* < 0.001) among four plant communities, ranging from 52 to 156 plant species (Fig. 4a, Supplementary data Table S3). PCV community (156 species) at lower altitudinal habitats (2568–2737 m) had the most plant species, followed by VPA (128 species) at the altitudinal range of 2952–3191 m. Furthermore, 58 plant species were found in the PAJ community at higher altitudes between 2874 and 3240 m. Furthermore, the AJP Community (52 species) at the medium altitudinal range of mixed coniferous forest had the lowest number of species (2989–3260 m). Species richness estimates in study sites varied from 32 to 87 species. Sampling site S87 had the most plant species (87), followed by sampling locations S84, S116, and S88, which had 86, 81, and 75 plant species, respectively. Furthermore, sampling site S128, which included 32 plant species, had the lowest number of species (Supplementary data Table S4).

The Shannon Diversity index (H’) values differed significantly (χ^2^ = 65.381, df = 3, *p* < 0.001) among four plant communities, ranging from 2.89 to 3.68 (Fig. 4b). Being recognized communities along the altitude, VPA community has the maximum value (H’ = 3.68) among all recorded communities followed by PCV (3.62), AJP (2.96) and PAJ (2.89; Fig. 4b).

The Simpson’s dominance index values differed significantly (χ^2^ = 60.289, df = 3, *p* < 0.001) among four plant communities, ranging 0.904–0.961 (Fig. 4c). After recognition of communities along the altitude, VPA and AJP communities each have the maximum value (0.961) followed by PCV community (0.947) and PAJ (0.904; Fig. 4c).

The Pielou’s evenness index values differed significantly (χ^2^ = 87.314, df = 3, *p* < 0.001) among four plant communities, ranging from 0.781 to 0.862 (Fig. 4d). Among the four plant communities recognized along altitude, VPA and AJP communities each have the maximum value (0.862) followed by PCV community (0.832). On the other hand, PAJ plant community was recorded with the lowest Pielous’s evenness value (0.781, Fig. 4d).

**Figure 5:**
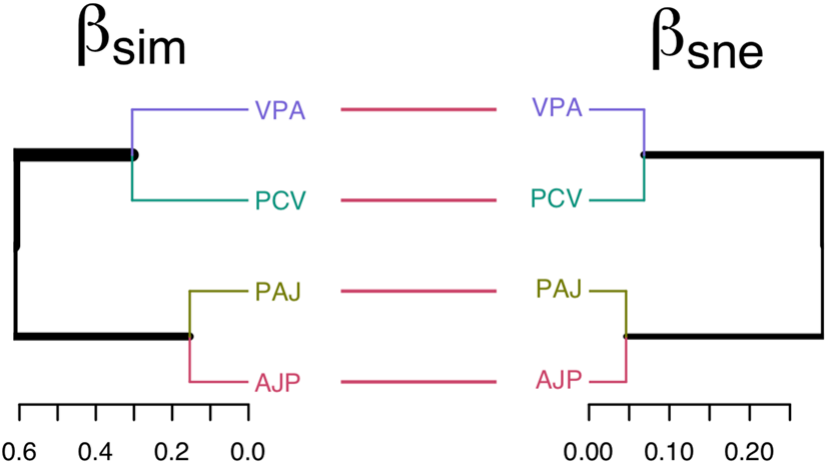
Dissimilarity cluster based on spatial turnover (βsim) and nestedness-resultant components (βsne) of beta diversity components of species dissimilarity between four plant communities of mixed coniferous forest. PCV, *Pinus-Cedrus-Viburnum*; VPA, *Viburnum-Pinus-Abies*; AJP, *Abies-Juniperus-Picea*; PAJ, *Picea-Abies-Juniperus.*

Spatial turnover (βsim) and nestedness-resultant components (βsne) of species dissimilarity revealed at least two distinct clusters (Fig. 5). In βsim cluster, we observed ~ 61% dissimilarity between AJP–PAJ cluster and VPA–PCV cluster. Also, VPA and PCV showed 30% of dissimilarity between them, while AJP and PAJ showed 15% dissimilarity. In βsne cluster, the highest dissimilarity value was 30% between the two clusters (AJP–PAJ and VPA–PCV). PAJ and AJP showed 5% dissimilarity, while VPA and PCV showed 6.8%. Thus, the spatial turnover of species (sim) has a greater impact on plant community structure than species loss (nestedness-resultant, sne).

### Variation of environmental variables among communities

**Figure 6:**
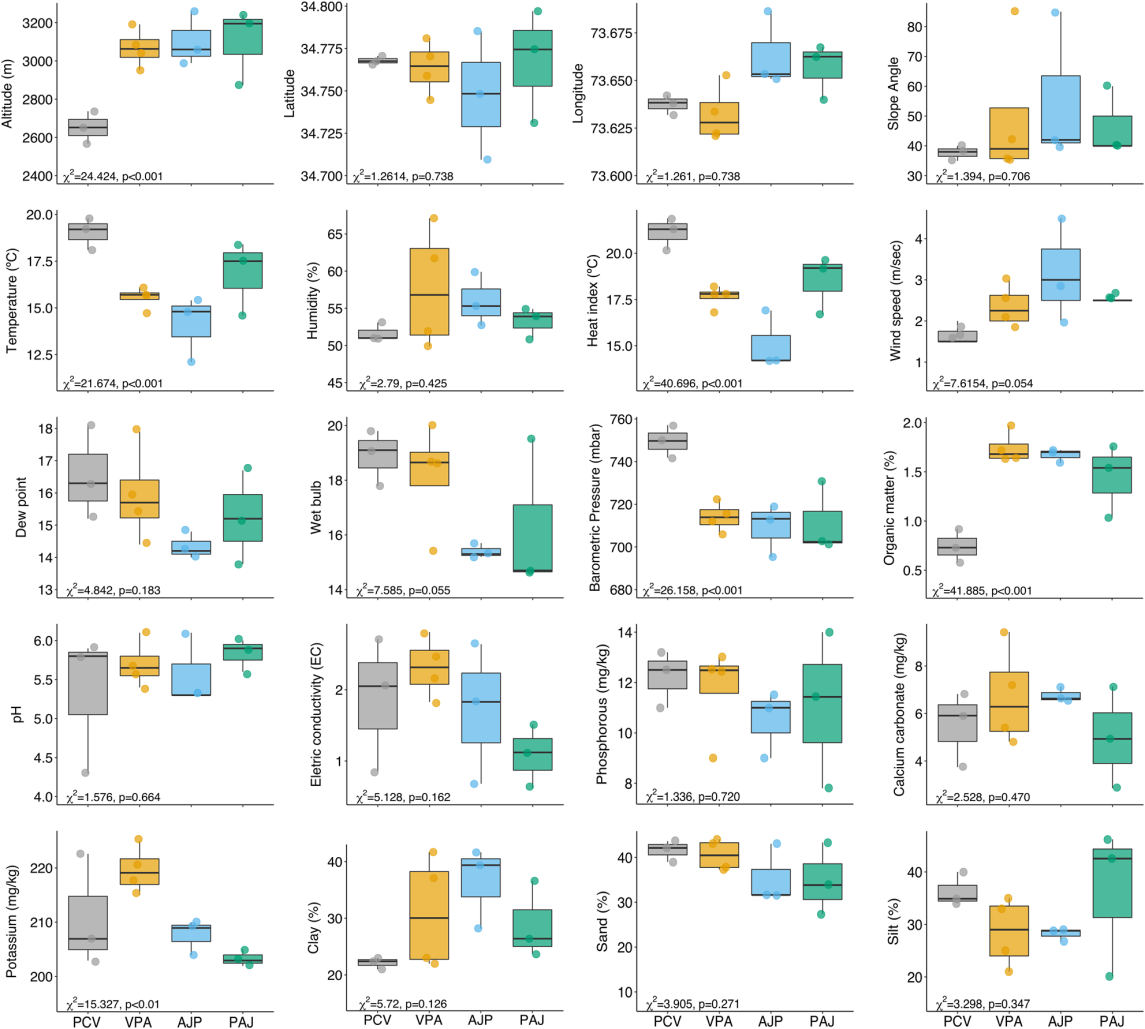
Boxplots showing the variations of the studied variables of the four plant communities evaluated in this study (GLM result, and its associated *p*-values are displayed at each whisker boxplot). Plant communities in x-axis were plotted in ascending order according to elevation gradient (low to high elevation). PCV, *Pinus-Cedrus-Viburnum*; VPA, *Viburnum-Pinus-Abies*; AJP, *Abies-Juniperus-Picea*; PAJ, *Picea-Abies-Juniperus.*

Most of the gradients were substantially different (*p*-value 0.05; Fig. 6) in GLM analyses of the 21 examined variables arranged in distinct classes (geographic, slope, climatic, and edaphic gradients) across the four plant communities. The only variables that did not indicate a significant difference were latitude, longitude, slope angle, humidity, dew point, pH, electric conductivity, calcium carbonate, phosphorus, and soil texture (i.e., sand, silt, and clay).

### NMDS and PCA

The ordination diagrams such as NMDS and PCA were used to identify the relationship between mixed coniferous forest vegetation and environmental factors (Fig. 3a–e). In both ordinations, we can see that the environmental variables separate the sampling sites in the four communities already demonstrated and confirmed by TWINSPAN. In addition, most of the communities of mixed coniferous forest was resided by 20–50% of clay, sand, and silty soil texture (Fig. S1).

### CCA and Partial CCA

**Figure 7:**
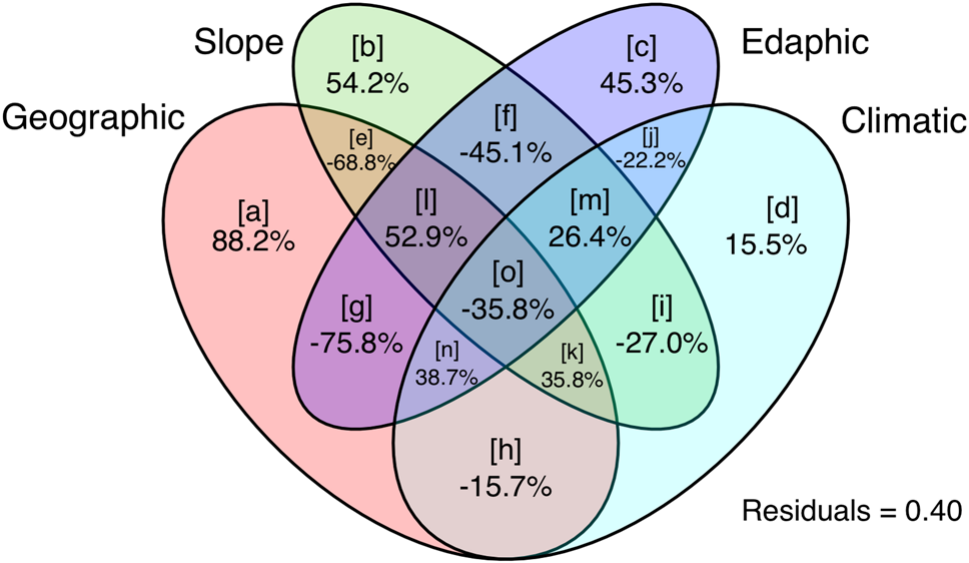
Variation partitioning findings (partial CCA) and contribution (percent) of the four variable groups analysed are shown in a Venn diagram. Negative values signify zeros, indicating that the explanatory factors explain less variance than random normal variables^82^.

Total inertia results of CCA were 1.695, where our final variables (pH, Slope aspect: NE and S, Humidity, Silt, Slope angle, Temperature, CaCO3, Longitude, Altitude) together explained 92.7% of variation (sum of canonical eigenvalues was 1.571). The first two canonical axes explained 51.6% of variation. CCA model was significant (pseudo-F value = 1.571; *p* < 0.005; df = 10; permutations = 999). For the 27 explanatory variables, we tested simple term effects. Simple term effects showed that Longitude, Temperature, pH, Slope aspect, S, and Altitude (decreasing order of importance) were significant (*p* < 0.05; Table 1). The 27 explanatory variables were grouped into four classes: Climatic (Humidity, Temperature, Dew point, Wet bulb, Wind speed, Heat index, Barometric pressure); Edaphic (pH, CaCO_3_, Phosphorous, Potassium, Organic matter, Electric conductivity, Sand, Silt, Clay, Clay loam, Loam, Silt loam); Geographic (Longitude, Latitude, Altitude); and Slope (Slope aspect: N, NE and S, Slope angle), and then, we performed variation partitioning tests (partial CCA) for all 15 possible classes (Table 2). Class [a] was the most explanatory variable (88.22%), followed by class [b] (54.27%), [l] (52.92%) and class [c] (45.37%) (Fig. 7).

**Table 1:** The contribution and ranking of the environmental variables used in our CCA model.

Ecological attributes	Df	Chi-square	F	Pr(> F)
Longitude	1	0.303993	49.223	0.003
Temperature	1	0.224163	36.297	0.013
pH	1	0.188659	30.548	0.011
Slope S	1	0.162332	26.285	0.017
Slope.NE	1	0.146564	23.732	0.059
Altitude	1	0.141325	22.884	0.045
Humidity	1	0.122259	19.796	0.127
Slope angle	1	0.111022	17.977	0.108
Silt	1	0.103366	16.737	0.152
CaCO_3_	1	0.068297	11.059	0.382

**Table 2:** Results of variation partitioning (partial CCA) of four variable groups studied (see Fig. 7 for individual fraction letters code).

Individual fraction	Adjusted R^2^	Variation explained (%)	% of all	Df
[a]	0.529	88.227	31.221	2
[b]	0.326	54.275	19.207	3
[c]	0.272	45.377	16.058	3
[d]	0.093	15.512	5.489	2
[e]	− 0.413	− 68.800	− 24.347	0
[f]	− 0.271	− 45.165	− 15.983	0
[g]	− 0.455	− 75.820	− 26.831	0
[h]	− 0.095	− 15.780	− 5.584	0
[i]	− 0.162	− 27.048	− 9.572	0
[j]	− 0.133	− 22.202	− 7.857	0
[k]	0.215	35.845	12.685	0
[l]	0.318	52.922	18.728	0
[m]	0.159	26.483	9.372	0
[n]	0.233	38.798	13.730	0
[o]	− 0.215	− 35.817	− 12.675	0
Total explained	0.600	100	35.437	10
All variation	1.696	/	100

## Discussion

It has long been the goal of ecological studies is to disentangle the dynamics that underlie the spatiotemporal distribution of biodiversity^83^, and further functions of the ecosystem^84,85^. Both biodiversity and ecosystem functions are driven by specific drivers of contemporary environments, i.e., biotic^86^ and abiotic variables^87–89^. Understanding how community composition varies in response to environmental variability is important in order to understand biodiversity^90^, productivity, and ecological stability^91,92^. With the advent of modern methods and techniques^93^, a number of contributions to modern plant ecology have addressed the question of complex vegetation patterns^70,94–96^. TWINSPAN categorized 218 plant species from 13 Manoor Valley mixed coniferous forest study sites into four primary plant communities. When such formation of ecological linkages is based on indicator values^97,98^, each plant community often contains one or more indicator species^99,100^. As the study area lies in the Himalayan region, the vegetation predominantly exhibited characteristics of Sino-Japanese nature. The communities were classified within this region were categorized based on a range of environmental gradients i.e., soil pH, organic matter, electrical conductivity, nitrogen, potassium, phosphorous contents, soil texture, slope angle and aspect, and altitude etc. This allows our results to be compared with the studies already undertaken in other adjacent Himalayan regions^101^. Our findings are consistent with those of^102^, who identified four plant associations during an eco-floristic investigation of Beer Hills along the Indus River in Pakistan. Similarly^103^, documented seven plant communities and five major forest types while studying phytosociological analysis of Western Himalayan forests of Muzaffarabad, Azad and Jammu Kashmir, Pakistan. 

Nonetheless, the current study plant communities were structured by the influence of various ecological variables like geographic, slope, edaphic and climatic gradients. It also revealed that the indicator species of each plant community were linked to the particular set of environmental gradients. A region's forest communities evolve through time, but altitude, slope, latitude, aspect, precipitation, and humidity all have a part in their development and composition^104–106^. Ecosystems respond to several simultaneous changes in the environment^107^, which affect community diversity and distribution^94,108^. In each microhabitat type, specialist plant communities thrive, and are composed of specific taxa that have adapted to the unique environmental conditions of that particular microhabitat^109^. Aboveground^28^ and underground communities work together to control whole-ecosystem processes and reactions to changes in the environment^110,111^.

The study area is richly diverse with Shannon Diversity index (H’) values ranged between 2.89 and 3.68. Among recognized communities along the altitude and other gradients, VPA community has the maximum value (H’ = 3.68) among all recorded communities followed by PCV community (3.62) and AJP (2.96). This bimodal response of diversity measures might be due to the anthropogenic pressure^112^. The higher anthropogenic activities probably be linked with the easy access to the such sites^113^. Furthermore, most of the communities of mixed coniferous forest were recorded on the sloppy surfaces and edges of the mountains that might be the reason of their diversity. Gehlhausen et al.^114^ observed the forest edges with maximum diversity as compared to the forest interiors. Similar pattern was reported by Khan^51^ from the neighbor valley (Naran Valley, Northwestern Himalaya). Such diverse species also reveals their wider ecological amplitude.

In the topographic class, we showed that altitudinal gradients offer an important range of different environmental variables, highlighting the existence of micro-climates that drive the structure and composition of plant species in each micro-region. We compared our results with other vegetation surveys carried out in allied^51^ and neighboring Himalayan regions^115–117^ which similarly described the influential role of altitude. As a result, the northern slopes sustained the denser and homogenous growth of conifers in Manoor Valley, Himalayan forests, Pakistan. In addition, the scores of variation partitioning indicated that topography was the main driver in mixed coniferous forests, whereas those results were found according to the findings of^118^.

The ordination study demonstrated that varied edaphic gradients had a substantial impact on the mixed coniferous forests, which were categorized into four primary plant communities. The structure of communities and plant growth, ground cover, and natural regeneration capabilities are all affected by edaphic gradients^119^. Likewise, the most influential contributing gradients were soil texture (loam, silty loam, clay and clay loamy), organic matter and potassium. In the present study, the loamy soil with high organic matter significantly contributed in structuring VPA community. Similarly, Dvorský et al.^116^ reported the contribution of soil moisture as the influential variable for shaping the community structure. This study depicted that the vegetation of mixed coniferous forests was strongly influenced by soil texture gradient and their contribution assessment was visualized by the direct ordination approaches. For instance, the PCV community was recorded under the influence of silty loam soil texture. Nonetheless, studies of^115,116^ have reported similar findings, they also concluded that soil texture was a strong contributing gradient in structuring the vegetation from the same terrain of Himalayas.

The results showed non-significant differences among communities in relation to electric conductivity, pH and phosphorous, but slight variations were noticed in their average means. At the higher altitudinal sites, maximum humification was recorded and that might be due to the higher cover which escorted to a drop-in soil pH. The lowest soil pH at higher elevations may further led to an increase in phosphorus content through mineralization. Current results were compared with the previous reports on Himachal Pradesh (Northwestern Himalaya), India^117^, which revealed similar findings by stating non-significant impact of soil pH and phosphorus on the vegetation due to high elevation. Lastly, the possible reasons of all these resemblances may be just because of the matched environmental conditions hosted by the mountains of Himalayan region that in turn governs the biotic and abiotic variables.

Finally, beta-diversity based on the turnover of species is the trait that most influence the distribution of plant species in the Himalayan mixed coniferous forest of Pakistan. In other words, instead of decreasing the number of species along the altitudinal gradient (used as a proxy for climatic and other environmental gradients^120,121^) and under different climatic conditions, there is a species turnover, i.e., plants that live in high elevations might have different traits than those living in low elevations^122,123^. This variation might have allowed them to survive in this harsh or different environments, leading to a variation in plant community along the altitude. Changes in the environment alter the diversity and organization of plant communities by altering the spectrum of species features that may be effective in new environments^124^.

In addition to geographic, topographic, and edaphic gradients, climatic gradients also represented a vital role in hosting the major plant communities of mixed coniferous forests. The most significant contributing gradients were temperature, heat index, wind speed, wet bulb, and barometric pressure. The analytical approaches revealed the positive and significant correlation of PCV community with temperature, heat index and barometric pressure. This might be due to the region hosting this community were located at the lower altitudinal ranges as compared to other major groups. As we all know, the response of vegetation structure to changes in environmental gradients has a significant impact on its development. Among all the recorded plant communities, PCV community was the dominant one based on the number of associated plant species (156 species). This variation in the number of associated plant species within communities might be due to variability in the values of edaphic and other environmental gradients^125,126^ which are responsible in sustaining the growth of various associated species^127^. Plant communities can be described in a way that assists management decisions for a variety of ecological communities^128^.

## Conclusions

Multivariate analyses categorized 218 plant species found at 13 study sites in the mixed coniferous forest into four distinct plant communities, each with its own indicator species. Among all the recorded plant communities, PCV community was the dominant one based on the number of associated plant species (156 species). The impact of numerous ecological factors shaped these plant assemblages. In the topographic class, altitude was shown to be the most important gradient, followed by latitude and longitude. The northern slopes fostered the denser and more uniform development of conifers in Manoor Valley, Himalayan forests, Pakistan. In addition, the scores of variation partitioning indicated that topography was the main driver in mixed coniferous forests. The most influential contributing edaphic gradients were soil texture (loam, silty loam, clay and clay loamy), organic matter and potassium. Likewise, the most significant contributing environmental gradients in structuring and hosting the plant communities of mixed coniferous forest were temperature, heat index, wind speed, wet bulb, and barometric pressure. The topmost indicator species and other associates of VPA community was hosted by loamy soil with higher sandy texture, calcium carbonate and OM as compared to other communities. These indicator species could be used to observe changes in plant communities as a result of changes in the environment or management. As a result, recognizing such an indication might be used to manage species in a range of microhabitats with varying soil types and climatic conditions.

Assessing the abiotic and biotic variables that drive the ecosystem dynamics is one of the main goals nowadays, mainly due to the continuous process of climate change and anthropogenic impacts. Studies like this can help in understanding the structure of plant communities, observing how each community responds to a certain environmental change. In this context, it is possible to identify (1) indicator species, (2) anthropic impacts, and (3) climate and soil changes in certain environments, or even provide mitigating and preventive measures to reduce these impacts. Finally, the Himalayas, where this study was developed, is a highly biodiverse region with high endemism and degradation, being classified as a biodiversity hotspot^129^. Studies evaluating the structure of plant communities residing in these locations are therefore essential for maintaining biodiversity.

### Supplementary Information

Supplementary Figure S1.

Supplementary Tables.

## Data Availability

All data related to this article is presented here, as the data is not archived anywhere else.

## Abbreviations

IVI: Importance value index
EC: Electrical conductivity
OM: Organic matter
P: Phosphorus
K: Potassium
CaCO_3_: Calcium carbonate
TWINSPAN: Two-way indicator species analysis
PCA: Principle component analysis
CCA: Canonical correspondence analysis
VIF: Variance inflation factor
PCV: *Pinus-Cedrus-Viburnum*
VPA: *Viburnum-Pinus-Abies*
AJP: *Abies-Juniperus-Picea*
PAJ: *Picea-Abies-Juniperus*
