# Supplementary material for: Vegetation–environment interactions: plant species distribution and community assembly in mixed coniferous forests of Northwestern Himalayas
Source: Sci Rep. 2023 Oct 11;13:17228. doi: 10.1038/s41598-023-42272-1 (PMC10567734; doi:10.1038/s41598-023-42272-1)
Supplement: Supplementary file 1 — Supplementary Figure S1. [file 41598_2023_42272_MOESM1_ESM.docx]

**Vegetation–environment interactions: plant species distribution and community assembly in mixed coniferous forests of Northwestern Himalayas**

**Inayat Ur Rahman^1,2,3*^, Robbie E. Hart^2^, Aftab Afzal^1*^, Zafar Iqbal^1^, Rainer W. Bussmann^4,5^, Farhana Ijaz^1^, Muazzam Ali Khan^6^, Hamid Ali^7^, Siddiq Ur Rahman^8^, Abeer Hashem^9^, Elsayed Fathi Abd_Allah^10^, Ali Sher ^11^, Eduardo Soares Calixto^12,13^**

*^1^Department of Botany, Hazara University, Mansehra 21300, Khyber Pakhtunkhwa, Pakistan*

*^2^William L. Brown Center, Missouri Botanical Garden, P.O. Box 299, St. Louis, MO 63166-0299, USA*

*^3^Department of Botany, Khushal Khan Khattak University, Karak 27200, Khyber Pakhtunkhwa, Pakistan*

*^4^Department of Ethnobotany, Institute of Botany, Ilia State University, 1 Botanical Street, Tbilisi 0105, Georgia*

*^5^Department of Botany, State Museum of Natural History, Karlsruhe, Germany*

*^6^Department of Botany, Bacha Khan University, Charsadda 24460, KP, Pakistan*

*^7^Department of Biotechnology & Genetic Engineering, Hazara University, Mansehra 21300, KP, Pakistan*

*^8^Department of Computer Science & Bioinformatics, Khushal Khan Khattak University, Karak 27200, Khyber Pakhtunkhwa, Pakistan*

*^9^Botany and Microbiology Department, College of Science, King Saud University, P.O. Box. 2460, Riyadh 11451, Saudi Arabia*

*^10^Department of Plant Production, College of Food and Agriculture Science, King Saud University, Riyadh 11451, Saudi Arabia*

*^11^ Department of Botany, Qurtaba University Peshawar, KP, Pakistan*

*^12^Department of Biology, University of Missouri St. Louis (UMSL), Saint Louis, MO, USA*

*^13^Entomology and Nematology Department, University of Florida, Gainesville, FL, USA*

*Correspondence should be addressed to [driurahman@gmail.com](mailto:driurahman@gmail.com); aftabafzalkiani@yahoo.com

**Figure S1:** Distribution of 13 sampling sites based on their corresponding set of environmental gradients of mixed coniferous forests.
